# The role of perceived stress and cognitive function on the relationship between neuroticism and depression among the elderly: a structural equation model approach

**DOI:** 10.1186/s12888-020-2440-9

**Published:** 2020-01-20

**Authors:** Mukda Banjongrewadee, Nahathai Wongpakaran, Tinakon Wongpakaran, Tanyong Pipanmekaporn, Yodying Punjasawadwong, Sirirat Mueankwan

**Affiliations:** 1 Geriatric Psychiatry Unit, Department of Psychiatry, Faculty of Medicine, Chiang Mai University, 110 Intawaroros Rd., T. Sriphum, A. Muang, Chiang Mai, 50200 Thailand; 2 Department of Anesthesiology, Faculty of Medicine, Chiang Mai University, Chiang Mai, Thailand; 3 Division of Surgical Critical Care and Trauma, Department of Surgery, Maharaj Nakorn Chiang Mai Hospital, Chiang Mai, Thailand

## Abstract

**Background:**

Depression comprises common psychological problems, and has been strongly related to neuroticism and perceived stress. While neuroticism has been shown to have a direct effect on depression, it also has an indirect effect via perceived stress. Among the elderly, cognitive function produces influences that should not be overlooked when investigating depression. This study aimed to determine the role of mediating effects of perceived stress as well as cognitive function on neuroticism and depression among elderly patients.

**Methods:**

This research constituted a secondary analysis, with data collected during the pre-operative period of 429 elderly individuals undergoing elective, noncardiac surgery. The evaluation included the Perceived Stress Scale, the Neuroticism Inventory, the Montreal Cognitive Assessment, and the Geriatric Depression Scale. Structural equation modeling was used to investigate the hypothesized model.

**Results:**

Neuroticism exhibited a significant indirect effect on perceived stress via depression and cognition (β = 0.162, 95% CI 0.026, 0.322, *p* = .002). Neuroticism initially had a direct effect on depression (β = 0.766, 95% CI 0.675, 0.843 *p* = 0.003); thereafter, it was reduced after covariates were added (β = 0.557, 95% CI 0.432, 0.668 *p* = 0.002). Based on this model, the total variance explained by this model was 67%, and the model showed an acceptable fit with the data.

**Conclusions:**

Both perceived stress and cognitive function partially mediated the effect of neuroticism on depression, with perceived stress exhibiting a greater effect.

**Trial registration:**

The study protocol has been registered at Clinicaltrials.gov under registered number: NCT02131181.

## Background

Depression among the elderly is associated with various risk factors, including personality traits such as neuroticism. Neuroticism, which is one of the Big Five higher-order personality traits, represents the tendency to experience negative emotions, such as anxiety and anger, leading to susceptibility to psychological distress and vulnerability to stress [1]. Neuroticism is associated with negative emotions such as anxiety, fear, and anger [2].

There is a well-established relationship between neuroticism and depression, particularly among the elderly. Previous research has found that 15–25% of elderly persons experience high levels of neuroticism [3, 4].

On the one hand, the perception of stress following stressful life events conceivably precedes the development of depression, a process which is well-documented in the clinical literature [5–9]. *Perceived stress* involves the feelings or thoughts that individuals have regarding the extent of the *stress* they are experiencing at a given time. Perceived stress is significant for its biological, emotional, and physical consequences, including cardiovascular disorders, diseases related to poor immunological function, sleep problems and depression [8–18].

Perceived stress is one of the top five psychological health problems among elderly people in the US [19]. Among the elderly, perceived stress seems to be higher than it is in younger adults [20]. Relevant studies have revealed that the level of stress increases in relation to age in a linear fashion [20].

Perceived stress is closely related to neuroticism to the extent that they each constitute different facets [21]; however, a recent study suggested that perceived stress is not representative of neuroticism [22]. Neuroticism is one of the well-established predictors of perceived stress and depression [18, 23–26].

In terms of the relationship between neuroticism and perceived stress, higher neuroticism may promote negative emotional regulation or maladaptive reactivity to stress, which increases the predisposition to depressive symptoms. At the same time, studies have shown that perceived stress mediates the relationship between neuroticism and depression, serving to reduce the effect of neuroticism on depression [5, 27, 28]. However, both these studies were conducted in young and adult populations, rather than elderly participants. As such, there is a need for research addressing the relationship between these variables in elderly populations.

Cognitive function plays an important role in the interrelationships of neuroticism, depression, and perceived stress in elderly populations. The relationship between cognitive impairment and depression is well established [29]. Interestingly, perceived stress is related to lower initial cognitive scores and a faster rate of cognitive decline among adults aged 65 and above [30–33]. A recent study reported that perceived stress was a unique and modifiable risk factor for normal and pathological cognitive aging [34].

Another interesting relationship is that between cognition and neuroticism. Studies have demonstrated that high neuroticism is a risk factor for the development of cognitive impairment and dementia among the elderly [35–37]. Neuroticism is associated with cognitive function in most studies that use cross-sectional data. The results from longitudinal studies are inconsistent; nevertheless, majority of longitudinal studies (8/11) supported a relationship between neuroticism and cognitive function [38, 39].

Given the fact that cognitive function tends to decline with age, and the fact that the elderly are also more vulnerable to increased stress and neuroticism [39], elderly people may be particularly vulnerable to depression. There is an absence of studies including cognitive function in the mediation model of the relationship between depression, neuroticism, and perceived stress. It is not clear how these factors would come into play in depression among elderly people. To the best of our knowledge, no studies to date have addressed this issue.

In this study, we sought to investigate the relationship between these variables in a sample of elderly subjects. Moreover, we hypothesized that cognitive function would act as a mediator in the relationship between neuroticism and depression. Based on the reviewed evidence, we formed two hypotheses. First, both perceived stress and cognitive function would serve as mediators of the relationship between neuroticism and depression. Second, perceived stress would be associated with cognitive function, but more closely related to depression. We then hypothesized (in the multiple mediation model) that the indirect effect of neuroticism on depression via perceived stress would be characterized by a larger effect size than the indirect effect of neuroticism on depression via cognitive function.

## Materials and methods

This was a part of the study on the incidence of Post-operative delirium (POD) among 429 elderly individuals undergoing elective, noncardiac surgery. It was a cross-sectional analysis during their pre-operative period at a university hospital in northern Thailand between December 2013 and November 2014. An independent ethics committee approved this study’s ethics, and written informed consent forms were obtained from participants involved in the primary study. The study protocol has been registered at Clinicaltrials.gov under registered number: NCT02131181.

### Participants

The study included participants 60 years of age or older who were scheduled for elective noncardiac surgery. They were classified as American Society of Anesthesiologists (ASA) physical statuses I, II and III and provided informed consent. Older individuals with severe hearing/visual loss or dementia were excluded from the study. Those who were unable to communicate in Thai were also excluded. The participants provided information on the following: demographics, surgical diagnoses, surgery type, anesthetic technique, psychiatric/substance conditions and psychiatric assessments. The psychiatric assessments included the Perceived Stress Scale (PSS-10), the Neuroticism Inventory (NI), the Montreal Cognitive Assessment (MoCA) and the Geriatric Depression Scale (GDS-15). All measurements involved Thai versions. The diagnosis of dementia was made by psychiatrist investigators, while research assistants administered MoCA tests for all participants, and assisted in the process of participants’ administering self-reporting questionnaires. The present study excluded 80 participants due to the exclusion criteria. A total of 349 study subjects were included in the final analysis.

### Measurements

#### 10-item perceived stress scale (PSS-10)

This scale is a 10-item self-report using a 5-point Likert scale format (0 = never to 4 = very often), and the total score ranges from 0 to 40 [40]. Higher scores indicate greater perceived stress. The Thai version demonstrated good reliability and validity and has been widely used for adults; and specifically, for the elderly [41].

#### Neuroticism inventory (NI)

The NI is a dimensional measure of the neuroticism personality trait based on Eysenck’s five-factor model [23]. The NI, developed by Wongpakaran et al., consists of a self-rating scale that includes 15 items with a 0 to 4 Likert scale [42]. A higher score reflects a higher level of neuroticism. Cronbach’s alpha was 0.83. Its validity was similar to that of the Thai Depression Inventory, the State-Trait Anxiety Inventory, the Multi-dimensional Scale of Perceived Social Support (MSPSS) and the Inventory of Interpersonal Problems. Its correlation coefficients are 0. 0.61, − 0.23, 0.52, and 0.60, respectively (*p* < 0.001 for all).

#### Montreal cognitive assessment (MoCA)

The MoCA cognitive test evaluates 11 cognitive functions and the Thai version was used [43]. This test can be completed in 10 min [44]. The total score is 30, and the cut-off score for cognitive impairment is 25. Cronbach’s alpha coefficient was 0.74, Pearson’s correlation coefficient was 0.91, sensitivity was 70%, and specificity was 95% [45]. However, in ROC curve analysis to determine the optimal cut-off score of MoCA against Mental State Examination T10 (a modified Thai version of MMSE 2002) [46], the cut-off score 12/13 was suggested by Youden’s Index to detect dementia. This cut-off score yielded a sensitivity of 83.33 (95%CI, 70.7–92.40), and specificity of 82.40 (95%CI, 78.2–86.1). Area under the ROC curve (AUC) = 0.914 (0.0171)(95%CI, 0.884–0.939), with a *p* value <.0001. Therefore, 80 subjects, who scored MoCA below 13, were excluded for analysis.

#### The 15-item geriatric depression scale (GDS-15)

The GDS-15 is a widely used self-rating assessment tool that measures depressive symptoms among the elderly [47]. The Thai version shows a good internal consistency (Cronbach’s alpha was 0.85) [48].

### Statistical analyses

The scores of the PSS-10, NI, MoCA and GDS-15 were reported using descriptive statistics. The correlation between PSS-10, NI, MoCA, and GDS-15 was analyzed to confirm the association among variables, which was appropriate for a mediation model. IBM SPSS, Version 22 was used for this analysis.

To examine the relationship among variables, we used SEM comprising a measurement model and the structural model. SEM used latent variables to account for measurement error of the scales, i.e., of neuroticism, depression and perceived stress, and to specify the relationships among latent variables, while path analysis using observed variable (total score) assumes that all variables are measured without error. For MoCA, observe score was used as it was a composite score.

For the structural equation model (SEM), we created three parcels for neuroticism and two parcels for depression and PSS. We determined each as parcels 1, 2, or 3 according to the loading coefficients. We tested each measurement model and assessed the parcel and subscale loadings on the latent constructs before testing the SEM. The model fit was assessed using standard χ2 fit statistics, the Comparative Fit Index (CFI), the Tucker-Lewis index (TLI), and the root mean square error of approximation (RMSEA). A CFI and TLI greater than 0.95 and a RMSEA less than 0.06 indicated a good model fit. Because χ2 statistics are sensitive to sample size, we used the ratio of χ2/df < 3 as an acceptable model fit.

Because latent variables were used instead of observed variables, the latent mediation structural equation model was applied using full information maximum likelihood with robust standard errors. It was also used to assess the latent SEM. Data were checked for normality, outliers and multicollinearity before conducting CFA and SEM. A multiple imputation method was used to correct missing data. In each SEM, 21 parameters needed to be estimated. The ratio of sample size to the number of parameters to be estimated was approximately 20:1, which was acceptable. In approaches to investigate the mediating effect of depression and cognition on the relationship between neuroticism and perceived stress, the following significant correlations should be established: between neuroticism and cognition, between cognition (depression) and perceived stress and between neuroticism and perceived stress controlling cognition (depression) [49]. The total indirect effect of neuroticism on cognition and depression was analyzed. Age and sex were controlled for each mediation model and bias-corrected bootstrapping was applied for testing indirect effects.

## Results

The study consisted of 349 participants: 149 males (42.7%) and 200 females (57.3%). The mean age was 69.15 ± 6.38 years old. The mean educational attainment was 7.07 ± 5.16 years (median = 4, mode = 4). The vast majority reasons for surgery was malignant neoplasm (33.0%). The most common co-morbidity was hypertension (54.44%). The means of the PSS, NI, MoCA, and GDS-15 scores are shown in Table 1.

**Table 1:** Demographics and clinical characteristics of participants

Characteristics (*n* = 349)	Value n (%) or mean (SD)	Range
Sex, Female	200 (57.3)	
Age, years	69.15 (6.38)	60–86
Years of education	7.07 (5.16)	0–23
Main Physical illness for surgery		
Malignant neoplasm	115 (33.0)	
Primary gonarthrosis	34 (9.7)	
Calculus of gall bladder	32 (9.2)	
Fracture	20 (5.7)	
Benign neoplasm	19 (5.4)	
Inguinal hernia	13 (3.7)	
Uterovaginal prolapse	10 (2.9)	
Co-morbidities		
Hypertension	190 (54.44)	
Diabetes Mellitus	45 (12.89)	
Renal disease	41 (11.75)	
Chronic obstructive pulmonary disease	12 (3.44)	
Ischemic heart disease	21 (6.02)	
PSS-10	12.45 (5.33)	0–27
NI	30.87 (9.22)	15–57
MoCA	18.25 (3.71)	13–29
GDS-15	3.57 (2.29)	0–13

*Abbreviations*: *n* Number, *SD* Standardized deviation, *PSS-10* 10-item Perceived Stress Scale, *NI* Neuroticism inventory, *MoCA* Montreal Cognitive Assessment, *GDS-15* 15-item Geriatric Depression Scale

Pearson product moment correlation coefficients between each variable are presented in Table 2. As can be seen in Table 2, these correlation coefficients ranged from nil to moderate (.015–.610). Two points are worth mentioning regarding these correlations. First, a significant correlation was found between MoCA, GDS, NI, and PSS. The magnitude of the correlations between GDS and NI was the largest (*r* = .554, *p* < .001). MoCA had a significant negative correlation with GDS, NI, and PSS. Second, demographic data especially MoCA had significant correlation with the interested variables by that males had significant, albeit low, correlation with education, and education was related to MoCA, GDS, and NI. As expected, advancing age negatively correlated with cognitive function assessed by MoCA. Therefore, demographic data were accounted for in the SEM.

**Table 2:** Correlation matrix between variables

	Age	Sex	Education	MoCA	GDS	NI	PSS
Age	1						
Sex	-.031	1					
Education	-.096	.168^a^	1				
MoCA	-.199^a^	.100	.610^a^	1			
GDS	-.015	-.067	-.234^a^	-.305^a^	1		
NI	-.094	-.028	-.270^a^	-.293^a^	.554^a^	1	
PSS	-.018	-.033	-.089	-.195^a^	.453^a^	.363^a^	1

*PSS-10* 10-item Perceived Stress Scale, *NI* Neuroticism inventory, *MoCA* Montreal Cognitive Assessment, *GDS-15* 15-item Geriatric Depression Scale

^a^Correlation is significant at the 0.01 level (2-tailed)

### Testing for a mediation model

**Fig. 1:**
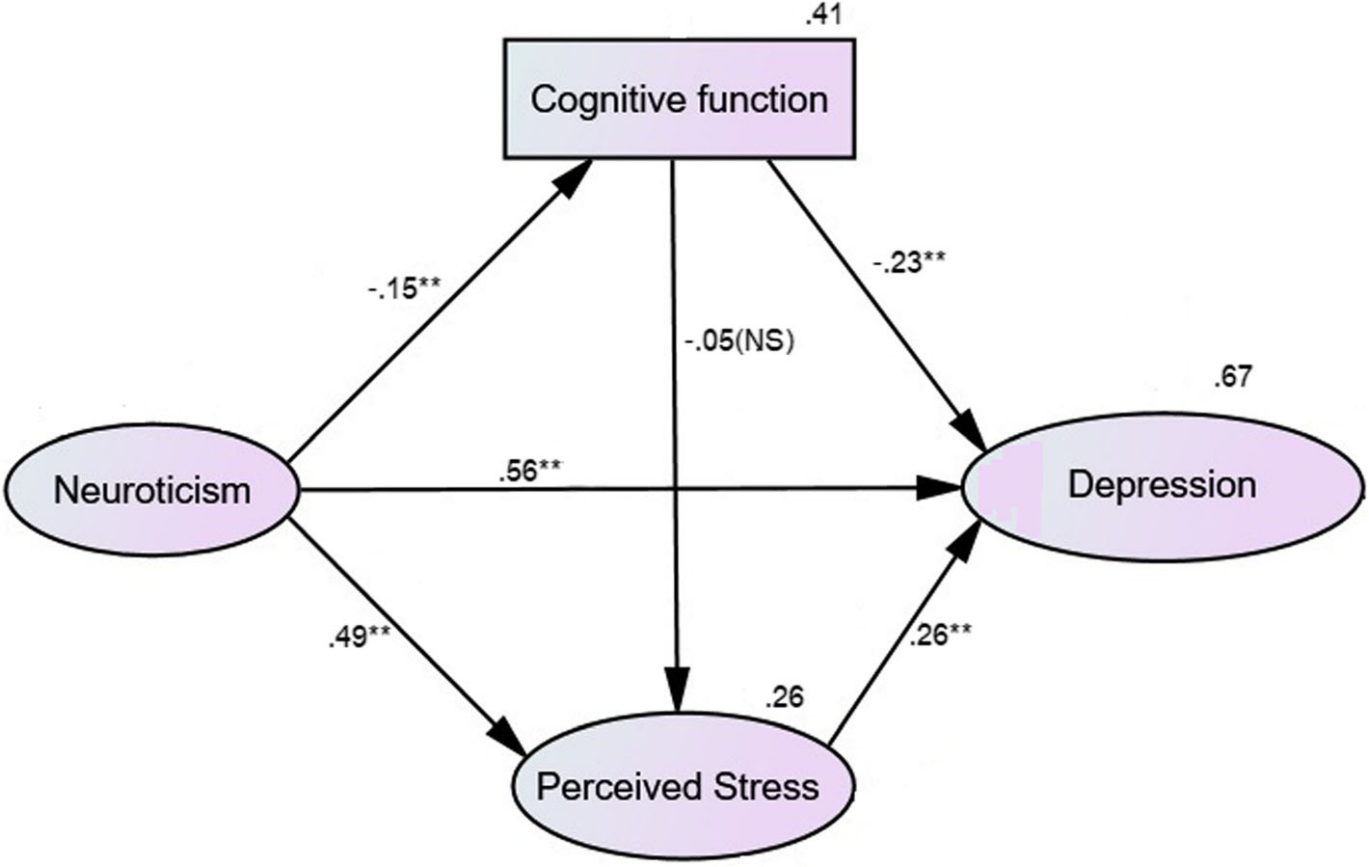
The standardized estimates of the hypothesized model. Legend: rectangular = observed variable, oval = latent variable, number on variable = R square value. *p < .05, **p < .01, NS = nonsignificant

After controlling for sex, age and education, the hypothesized multiple mediator model (Fig. 1) showed that neuroticism exhibited an indirect effect on depression via perceived stress and cognitive function (β = 0.162, 95% CI [0.026, 0.322], *p* = .002). Separately, neuroticism had a significant indirect effect on depression via cognition alone (β = 0.036, 95% CI [0.020, 0.063], *p* = 0.001), while neuroticism had a significant indirect effect on depression via perceived stress alone (β = 0.134, 95% CI [0.062, 0.210], *p* = 0.003). This supported our first hypothesis.

Overall, neuroticism initially had a direct effect on depression (β = 0.766, 95% CI [0.675, 0.843], *p* = 0.003), which was reduced (β = 0.557, 95% CI [0.432, 0.668], *p* = 0.002) after all covariates were held constant. However, the direct effect of cognitive function on perceived stress was non-significant (β = − 0.05, 95% CI [−.146, 0.036], *p* = 0.344).

The model demonstrated an acceptable fit for the data: CFI = 0.983, TLI = 0.972, RMSEA = 0.041 (90% CI [.017, .061]), χ^2^ = 52.35, df = 33, *p* = .017, χ^2^/df = 1.59.

In the decomposition of the model, 64.0% of the variance in perceived stress was accounted for when depressive symptoms served as a mediator. When cognition was added as the second mediator, this multiple mediator model explained 67.3% of the variance in depression.

As cognitive impairment may correlate with perceived stress and/or neuroticism, we also tested an alternative model by having cognitive function serve as a precursor of neuroticism, perceived stress and depression. Thus, cognitive function was treated as an exogenous variable, while neuroticism and perceived stress were treated as endogenous variables (only the path between cognitive function and perceived stress was oppositely redirected). The structural equation modeling analysis showed similar results to those hypothesized in the original model, for which chi-square was 52.39 (df = 33), CFI = .983, TLI = .972 and RMSEA 0.41 (90% CI [.018-, .061]). The extent to which these variables accounted for the variance in depression was the same (67%). However, the path coefficient between neuroticism and cognitive function became non-significant as it was mediated by perceived stress (β = 0.083, 95% CI [−.169, 0.010], *p* = 0.123). This supported our second hypothesis. Based on theory and empirical evidence, personality traits such as neuroticism do not change markedly during the course of life, while cognitive function does change as a result of age, stress and depression. Hence, the original hypothesized model was more suitable than the alternative model and became the final hypothesized model.

## Discussion

The present study investigated the extent to which neuroticism, perceived stress and cognitive function influenced depression. Both of our hypotheses were confirmed and, consistent with related studies on adults, we found that these variables had a significant effect in elderly people [27, 28, 50, 51]. That is, the present findings suggested that both perceived stress and cognitive function act as a mediator between neuroticism and depression in elderly people.

A few points from this study may serve to broaden our current knowledge. Firstly, by adding cognitive function to the multiple mediator model, the variance in depression can be better explained, suggesting that there is an effect of cognition on depression among the elderly. A recent study revealed that the association between neuroticism and cognitive function may be mediated by negative affect [39], and evidence indicated that negative affect was linked to perceived stress [52–54]. However, an extensive review by Curtis et al. found inconsistent results in regard to the relationship between neuroticism and cognition [38]. Most of the studies reporting a significant relationship between neuroticism and cognition using either cross-sectional or longitudinal data; some did not find such a relationship. It is interesting that the pattern in the hypothesized model (with a small but significant relationship between neuroticism and cognitive function) disappeared in the alternative model, where the path was redirected from perceived stress to cognition. The non-significant reverse models provide empirical support for the directionality of the hypothesized indirect effects of neuroticism via perceived distress on cognitive function. In other words, perceived stress mediated the relationship between neuroticism and cognition. This finding suggests that it is worth including perceived stress as a variable in further studies exploring the relationship between neuroticism and cognition, whether they be cross-sectional or longitudinal.

Previous studies have indicated the existence of the relationship between neuroticism, perceived stress, and depression in elderly people with clinical depressive disorder [55, 56], and the present study supports the existence of such relationships among elderly people without clinically diagnosed depressive disorders. This might apply to the elderly at similar pre-operative periods (or in related clinical settings) as a means of mitigating the severity of depressive symptom, heightened perceived stress, or cognitive decline following operations which typically lead to increased morbidity or prolonged hospitalization [57].

The final point we would like to highlight is that this multiple mediator model explained up to 67% of the variance in depression, while cognitive function explained only 3%. Although this effect may seem minimal, recall that we measured global cognitive function using the Montreal Cognitive Assessment (MoCA). Neuroticism may have had an effect on specific cognitive domains such as perceptual speed, working and episodic memory, and fluid ability [39, 58, 59], and this effect may be worth further investigation because the magnitude of association between neuroticism and some specific cognitive domain may vary. Furthermore, it would be ideal to conduct a longitudinal analysis, trait neuroticism is found to be relatively stable [60–62]. Hence, it could be posited as a precursor to be identified in the elderly in a clinical setting, while perceived stress and depression are less stable variables which are prone to change over time. Neuroticism may have temporal precedence over the other two variables [63]. Cognitive function, which appeared to be a mediator of the relationship between neuroticism and depression, is usually assessed in older adults. Poor cognitive function can worsen depressive symptoms in the elderly [64]. Cognitive stimulation is usually provided for patients with mild cognitive impairment, especially in geriatric clinics [29, 65]. However, as a predictor of depression, cognitive function is overshadowed by perceived stress. Clinicians should aim to plan for both cognitive improvement and stress reduction, particularly among those individuals who exhibit high levels of neuroticism at the screening phase or during the pre-operative period.

### Strengths and limitations

To the best of our knowledge, this is one of the first studies to assess for a relationship between depression, neuroticism, perceived stress and cognition in older adults. This study has several limitations. It was conducted with a small sample size, during a pre-operative period. Thus, the generalizability of this study’s findings to the entire elderly population is limited. Replications of this study in the general population or for a specific population of interest are strongly encouraged. Due to its cross-sectional design, this study cannot demonstrate any cause-effect relationships. However, this snapshot of cross-sectional data paves the way for a longitudinal data analysis. Moreover, these cross-sectional results could set the stage for a more carefully delineated design addressing some unanswered questions, including the impact of different types of cognitive function on depression.

Lastly, depression scores from self-report geriatric depression scales may not represent clinical depression as diagnosed by the DSM-5. In future studies, clinical diagnoses should be used to provide more clinical benefit.

## Data Availability

The datasets generated and/or analysed during the current study are not publicly available due to ethics approval but are available from the corresponding author on reasonable request.

## Abbreviations

CFI: Comparative Fit Index
GDS: Geriatric depression scale
MoCA: Montreal cognitive assessment
MSPSS: Multi-dimensional Scale of Perceived Social Support
NI: Neuroticism inventory
POD: Post-operative delirium
PSS: Perceived Stress Scale
RMSEA: Root mean square error of approximation
SEM: Structural equation modeling
TLI: Tucker-Lewis index
